# N-Terminal Gly_224_–Gly_411_ Domain in Listeria Adhesion Protein Interacts with Host Receptor Hsp60

**DOI:** 10.1371/journal.pone.0020694

**Published:** 2011-06-29

**Authors:** Balamurugan Jagadeesan, Amy E. Fleishman Littlejohn, Mary Anne Roshni Amalaradjou, Atul K. Singh, Krishna K. Mishra, David La, Daisuke Kihara, Arun K. Bhunia

**Affiliations:** 1 Molecular Food Microbiology Laboratory, Department of Food Science, Purdue University, West Lafayette, Indiana, United States of America; 2 Purdue University Interdisciplinary Life Sciences Program, Purdue University, West Lafayette, Indiana, United States of America; 3 Department of Biological Sciences, Purdue University, West Lafayette, Indiana, United States of America; 4 Department of Computer Science, Purdue University, West Lafayette, Indiana, United States of America; 5 Department of Comparative Pathobiology, Purdue University, West Lafayette, Indiana, United States of America; University of California Merced, United States of America

## Abstract

**Background:**

Listeria adhesion protein (LAP) is a housekeeping bifunctional enzyme consisting of N-terminal acetaldehyde dehydrogenase (ALDH) and C-terminal alcohol dehydrogenase (ADH). It aids *Listeria monocytogenes* in crossing the epithelial barrier through a paracellular route by interacting with its host receptor, heat shock protein 60 (Hsp60). To gain insight into the binding interaction between LAP and Hsp60, LAP subdomain(s) participating in the Hsp60 interaction were investigated.

**Methods:**

Using a ModBase structural model, LAP was divided into 4 putative subdomains: the ALDH region contains N1 (Met_1_–Pro_223_) and N2 (Gly_224_–Gly_411_), and the ADH region contains C1 (Gly_412_–Val_648_) and C2 (Pro_649_–Val_866_). Each subdomain was cloned and overexpressed in *Escherichia coli* and purified. Purified subdomains were used in ligand overlay, immunofluorescence, and bead-based epithelial cell adhesion assays to analyze each domain's affinity toward Hsp60 protein or human ileocecal epithelial HCT-8 cells.

**Results:**

The N2 subdomain exhibited the greatest affinity for Hsp60 with a *K*_D_ of 9.50±2.6 nM. The *K*_D_ of full-length LAP (7.2±0.5 nM) to Hsp60 was comparable to the N2 value. Microspheres (1 µm diameter) coated with N2 subdomain showed significantly (*P*<0.05) higher binding to HCT-8 cells than beads coated with other subdomains and this binding was inhibited when HCT-8 cells were pretreated with anti-Hsp60 antibody to specifically block epithelial Hsp60. Furthermore, HCT-8 cells pretreated with purified N2 subdomain also reduced *L. monocytogenes* adhesion by about 4 log confirming its involvement in interaction with epithelial cells.

**Conclusion:**

These data indicate that the N2 subdomain in the LAP ALDH domain is critical in initiating interaction with mammalian cell receptor Hsp60 providing insight into the molecular mechanism of pathogenesis for the development of potential anti-listerial control strategies.

## Introduction

*Listeria monocytogenes* express multiple surface-associated virulence factors, which play significant roles in breaching the intestinal, blood–brain, and fetoplacental barrier to cause gastroenteritis, septicemia, meningitis, and spontaneous abortion [1], [2]. Uptake of the bacterium into nonphagocytic intestinal epithelial cells is mediated by multiple virulence factors such as Internalin A (InlA) and Internalin B (InlB), leading to pathogen adhesion and subsequent invasion [3]. InlA is a bacterial cell wall–anchored virulence protein that interacts with the host E-cadherin (E-cad) [4], [5]. Binding of InlA to E-cad leads to downstream signaling events triggering epithelial cell architecture alteration through actin polymerization, resulting in pathogen uptake [3], [6]. InlB plays a critical role in infection dissemination to host tissues by mediating bacterial invasion into epithelial, endothelial, hepatocyte, and fibroblast-like cells [6], [7], [8]. Met receptor tyrosine kinase (RTK), gC1qR, and heparin sulfate proteoglycans of host cells act as receptors for InlB [9], [10]. Involvement of both InlA and InlB in causing materno-fetal infection in gerbils and knock-in mouse line expressing E-cad has been proposed [11].

Additionally, several other virulence factors serve as adhesion and/or invasion factors, including Internalin J [12], virulence invasion protein, Vip [13], autolysin amidase, Ami [14], fibronectin binding protein [15], [16], lipoteichoic acid [17], a cysteine transporter protein, CtaP [18], and LapB [19]. Among these, structural details and protein domain functions of only InlA and InlB have been thoroughly investigated [3].

The internalin family of proteins contain many N-terminal leucine-rich repeat regions (LRRs) formed by tandem repeats, which are capable of engaging in protein–protein interactions [20]. The LRR domain of InlA consists of 22-amino-acid residue repeat domains forming a right-handed solenoid structure. Individual repeats are formed by an initial beta strand followed by 5 residues. These strands combine to form a 16-stranded beta sheet, creating a cavity for interaction with the EC-1 domain at the N-terminal of E-cad [5]. Additionally, a proline in position 16 of E-cad is required for the hydrophobic interaction between InlA and E-cad [21]. The presence of a glutamate at position 16 of E-cad is seen in some species, such as mouse, and prevents interaction with InlA; the glutamate residue creates a hydrophilic environment, resulting in unfavorable conditions for InlA interaction [21]. In addition to the LRR domain, the inter-repeat region downstream of LRR has been shown to be critical for binding to its receptor, E-cad [5], [22]. InlA also contains a C-terminal LPXTG motif for anchoring the protein to the bacterial cell wall [23].

*Listeria* adhesion protein (LAP) is an adhesion factor that plays an important role during the intestinal phase of *L. monocytogenes* infection [24], [25]. LAP is present in all *Listeria* spp. including two newly reported listeriae; *L. marthii* and *L. rocourtiae* (unpublished) but absent in *L. grayi*. It functions as a housekeeping enzyme, an alcohol acetaldehyde dehydrogenase (AAD; *lmo 1634*) consisting of 866 amino acids with an N-terminal acetaldehyde dehydrogenase (ALDH), and a C-terminal alcohol dehydrogenase (ADH) [26]. A putative NAD^+^ binding domain is located between Gly_427_–Gly_432_, and an Fe_2_^+^ binding domain is located between Gly_724_–Gly_742_ [27]. Although it is present in both pathogenic and nonpathogenic *Listeria* species, only pathogenic bacteria secrete and reassociate LAP onto the cell surface to promote host cell interaction [27], [28]. LAP belongs to a family of anchorless adhesins involved in mammalian cell adhesion [27], [29]. The host cell receptor is a mitochondrial chaperonin called heat shock protein 60 (Hsp60) [30]. We recently showed that LAP–Hsp60 interaction promotes transepithelial translocation of *L. monocytogenes* through a paracellular route [31], suggesting an alternate strategy for bacteria to cross epithelial barriers during the intestinal phase of infection. Furthermore, *L. monocytogenes* infection at low dosage also increases Hsp60 expression, promoting enhanced LAP-mediated epithelial translocation [31].

Our goal is to understand molecular and cellular mechanisms involved in the interaction between *L. monocytogenes* and intestinal epithelial cells. Thus, our objective in this study is to determine the LAP domain critical for host cell receptor interaction, using purified Hsp60 and cultured human ileocecal epithelial HCT-8 cells. Understanding the LAP-Hsp60 interaction at the protein domain level provides insight regarding the molecular mechanism of pathogenesis for the development of potential anti-listerial control strategies.

## Results

### LAP Structure and Domain Prediction

**Figure 1:**
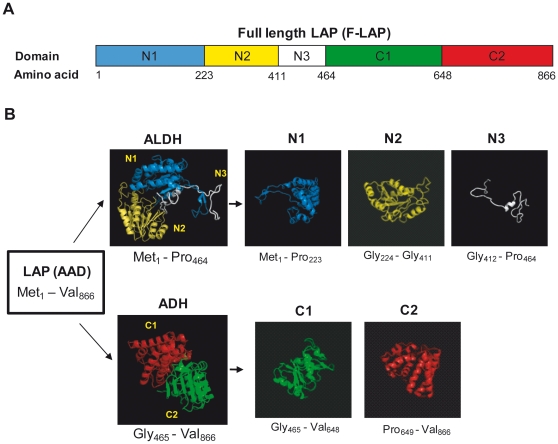
Listeria adhesion protein (LAP) structure predicted from homology modeling (ModBase). (**A**) LAP is an alcohol acetaldehyde dehydrogenase enzyme (AAD; 866 aa) and consists of an N-terminal acetaldehyde dehydrogenase (ALDH) and a C-terminal alcohol dehydrogenase region (ADH). The ALDH and ADH domains are modeled separately. The C terminus also contains an iron binding domain. (**B**) LAP is composed of alpha helices, beta sheets, and loop structures. N-terminal ALDH was determined to be composed of 3 independent subdomains: N1 (Met_1_–Pro_223_), N2 (Gly_224_–Gly_411_), and N3 (Gly_412_–Pro_464_). Similarly, C-terminal ADH was defined as having 2 independent subdomains: C1 (Pro_465_–Val_648_) and C2 (Pro_649_–Val_866_). N3 is a loop region that connects the LAP N- and C-terminus and is thought to be incapable of independent Hsp60 interaction; hence, this region was cloned and expressed in conjunction with the C1 subdomain. The N1, N2, N3, C1, and C2 subdomains are colored in blue, yellow, white, green, and red, respectively. Also using the same color scheme, the bottom panel represents the full LAP sequence and partitioned into the colored subdomains (boxed and labeled by the ending amino acid positions for each subdomain).

LAP is composed of five subdomains as schematically represented in figure 1A. It predominantly consists of alpha helices along with beta sheets and loop regions (Fig. 1B). Overall structure of LAP is not solved yet; however, LAP structural domains were predicted based on a structure model derived from ModBase (http://modbase.compbio.ucsf.edu/modbase-cgi/index.cgi), a database of protein homology models [32]. Five structural domains were identified by structure model: N1 (Met_1_–Pro_223_), N2 (Gly_224_–Gly_411_), N3 (Gly_412_–Pro_464_), C1 (Pro_465_–Val_648_), and C2 (Pro_649_–Val_866_) (Fig. 1B). The predicted structure of N3 (52 amino acids) shows little secondary structure, indicating that the purified protein may be unstable. Therefore, we expressed the N3 with C1 and revised the domain boundaries as follows: N1 (Met_1_–Pro_223_), N2 (Gly_224_–Gly_411_), C1 (Gly_412_–Val_648_), and C2 (Pro_649_–Val_866_).

### Cloning and Expression of LAP Subdomains

**Figure 2:**
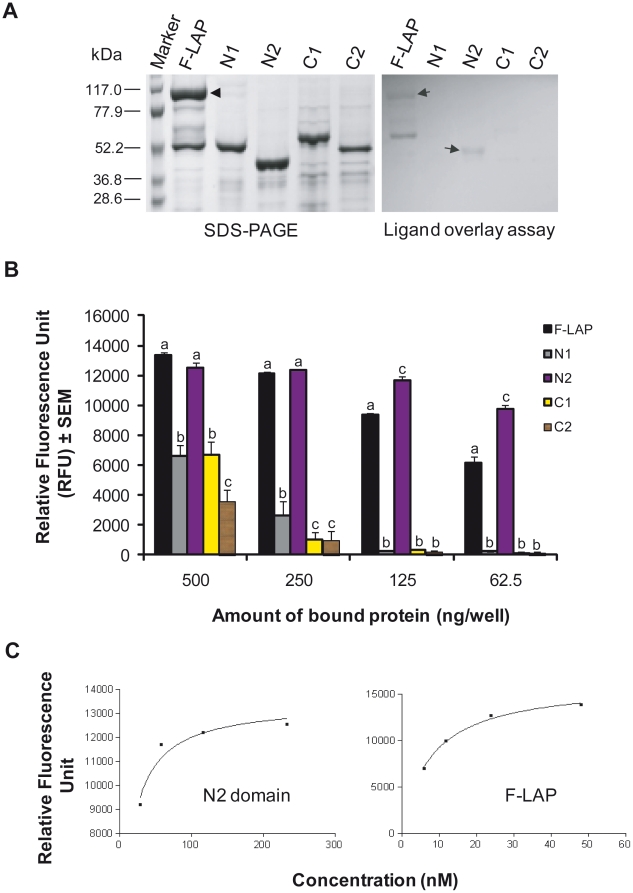
Analysis of LAP subdomain interaction with Hsp60. (**A;** left panel) SDS-PAGE (7.5% acrylamide) gel showing affinity-purified LAP subdomains. All purified proteins showed expected band size of approximately 45–54 kDa, which includes LAP subdomains; N1 (24 kDa), N2 (21 kDa), C1 (28 kDa) and C2 (26 kDa) and endogenous His, S and Trx tags derived from pET-32a cloning vector (Novagen). (**A,** right panel) represents ligand overlay assay showing purified Hsp60 binding to the N2 subdomain (arrow). As a positive control, full length LAP (F-LAP) also reacted with Hsp60 but showing two bands, second 53 kDa band is thought to be a breakdown product. (**B**) Immunofluorescence assay of LAP subdomains (N1, N2, C1, and C2) and full-length LAP (F-LAP) interaction with Hsp60. Of the 4 varying concentrations tested, the N2 subdomain showed significantly higher Hsp60 interaction (*P*<0.05) relative to other LAP subdomains. Values labeled with different letters (a, b, c) for a given protein concentration are significantly different at P<0.05.(**C**) Interaction of N2 and F-LAP with Hsp60 using values from 2B above. A logarithmic fitting curve was observed when values for each subdomain were plotted against their respective concentrations. An R^2^ value of greater than 0.9 was used for curve fitting. Binding affinity (*K*_D_) values for N2 and F-LAP interactions with Hsp60 were calculated using the 1-site binding equation in Graphpad. *K*_D_ values are the average of 2 independent curve-fitting experiments and were estimated to be 9.51±2.59 nM and 7.17±0.53 nM for N2 and F-LAP, respectively (average ± standard error of the mean [SEM]).

PCR-amplified DNA regions encoding LAP subdomains N1, N2, C1, and C2 were cloned and expressed in *Escherichia coli* and purified by nickel affinity chromatography. SDS-PAGE analysis revealed that all purified proteins had an expected band size of approximately 45–54 kDa (Fig. 2A), which includes LAP subdomains; N1 (24 kDa), N2 (21 kDa), C1 (28 kDa) and C2 (26 kDa) and endogenous His, S and Trx tags derived from pET-32a cloning vector (Novagen). These data indicate that LAP subdomains can be independently and stably expressed.

### N2 Subdomain Shows Increased Affinity toward Receptor Hsp60

A ligand overlay assay with SDS-PAGE separated proteins on Immobilon P membrane demonstrated that among LAP subdomains, only N2 domain strongly interacts with Hsp60, producing a visible band (Fig. 2A; right panel). As a control, full length LAP (F-LAP) also showed positive reaction with Hsp60. Though, two positive bands (104 and 53 kDa) were observed with F-LAP, the second band could be the natural degraded product of purified LAP (Fig 2A). Nevertheless, this study indicates that N2 subdomain of LAP has a strong binding affinity toward Hsp60.

We then measured LAP subdomain binding affinity to Hsp60. This was accomplished using an immunofluorescence assay performed in microtiter plates. LAP subdomains were immobilized at varying concentrations (62.5–500 ng/100 µL). At all concentrations, the interaction of N2 with Hsp60 was highest among all subdomains tested (Fig. 2B). The greatest difference (*P*<0.05) in N2 binding to Hsp60 compared to the other subdomains was observed at concentrations between 62.5 and 125 ng/100 µL. Binding affinities (dissociation equilibrium constant, *K*_D_) of LAP subdomains to Hsp60 were calculated from the 1-site binding equation by using Prism 3 software (Graphpad). Interaction of N2 and F-LAP produced logarithmic curve fittings (R^2^>0.9) when binding responses were fitted against the respective concentrations (Fig. 2C). *K*_D_ values for the N2 subdomain to Hsp60 and F-LAP to Hsp60 were estimated to be 9.50±2.6 nM and 7.2±0.5 nM, respectively, consistent with our previous report [27]. Curve fit analysis for N1, C1, and C2 subdomain interactions with Hsp60 indicated nonspecific interactions; thus, *K*_D_ values could not be calculated for these subdomains. These data indicate that N2 subdomain (Gly_224_–Gly_411_) of ALDH region is responsible for the interaction of LAP to its mammalian receptor, Hsp60.

### Beads Coated with N2 Subdomain Show Increased Binding to HCT-8 Cells

**Figure 3:**
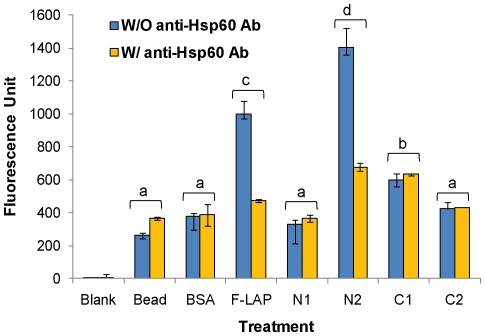
Bar graph showing binding of LAP subdomains-coated fluosphere (1 µm diameter) to human ileocecal epithelial HCT-8 cell monolayers. Among the subdomains, N2-coated fluospheres showed the most adherences compared to N1, C1, and C2. As a positive control, full-length LAP (F-LAP) - coated fluospheres also showed strong binding. Fluospheres alone (bead) and BSA-coated fluospheres (BSA) had also significantly (P<0.05) low binding. Pretreatment of HCT-8 monolayers with anti-Hsp60 MAb significantly (P<0.05) reduced the binding of N2-coated and F-LAP-coated fluospheres. Data are average of 3 independent experiments run in duplicate and presented with standard error of mean (SEM). Values labeled with different letters (a, b, c, d) are significantly different at P<0.05.

**Figure 4:**
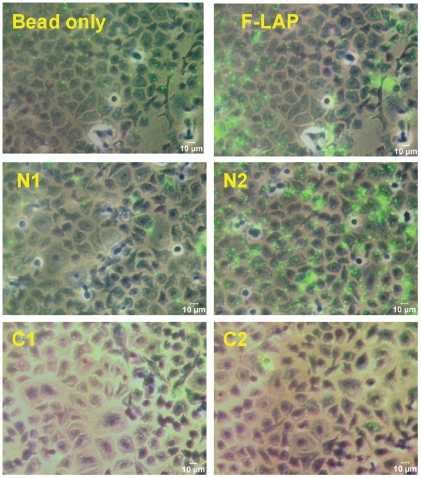
Representative photomicrographs from **Fig 3**, showing fluosphere binding to HCT-8 cell monolayers. Cell monolayers were counter stained with Giemsa stain before examining under Leica microscope (400X magnification). Cell monolayers exposed to N2 and F-LAP-coated fluospheres showed higher numbers of fluospheres compared to other treatments.

To demonstrate the biological relevance of LAP subdomains we analyzed binding of each subdomain using an *in vitro* cell culture model. Purified LAP subdomains were immobilized on sulphate-modified microsphere (fluospheres; 1.0 µm avg diameter, Invitrogen) with an average bound protein concentration of 140 µg/ml, exposed to human ileocecal epithelial HCT-8 cell monolayers, and adhesion was measured by spectrofluorometer [33]. Individual LAP subdomain-coated bead binding to HCT-8 cells from 3 independent experiments were analyzed (Fig. 3). N2-coated fluospheres showed most adherence to HCT-8 cells with mean fluorescence unit of 1401.4±120.6, followed by F-LAP-coated fluospheres (999.4±81.1) which were significantly (P<0.05) higher than C1 (597.2±26.4), C2 (423.8±36.9), and N1 (330.30±26.40) - coated fluospheres. Beads alone (262.4±18.6) and BSA-coated fluosphere (375.7±20.7) had also very low binding. Representative images of subdomain-coated fluosphere binding to HCT-8 cell monolayers are presented in Fig. 4. These results clearly demonstrate that N2 is the primary LAP subdomain responsible for epithelial cell binding with significantly higher binding (P<0.05) than any of the other domains.

To further confirm, if N2 subdomain interaction with host cell receptor, Hsp60 on epithelial cells is specific, we pretreated the HCT-8 cell monolayers with anti-Hsp60 monoclonal antibody for 1 h and then binding of protein coated fluospeheres was measured. Data show that among the subdomains, only N2- fluospheres binding to HCT-8 cells was significantly (P<0.05) reduced than the other sub-domains. As expected F-LAP coated fluospheres also showed reduced binding (Fig 3). These data again confirm that N2 interaction with Hsp60 on epithelial cells is highly specific, which could be blocked by anti-Hsp60 antibody.

### Inhibition of *L. monocytogenes* Adhesion to Epithelial Cells by Recombinant Proteins

**Figure 5:**
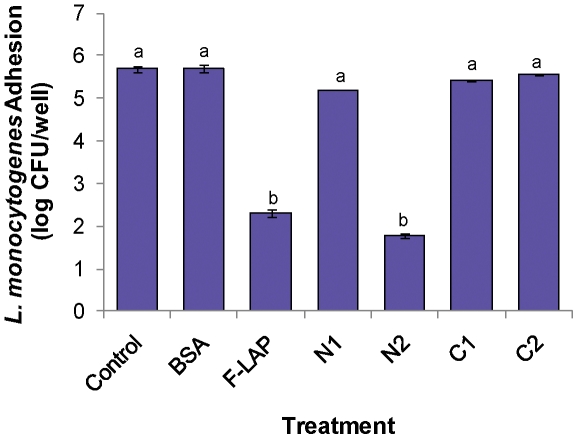
Adhesion analysis of *Listeria monocytogenes* cells to HCT-8 epithelial cells pretreated with LAP subdomain proteins (40 µg/well). N2 and F-LAP showed significant reduction (about 3-4 log cfu/ml) in *L. monocytogenes* adhesion to HCT-8 monolayers than the other treatments including BSA, N1, C1, C2 and untreated control. Bars marked with letters (a,b) are significantly different at P<.05.

To validate the direct involvement of N2-subdomain binding to epithelial cells, we pretreated the HCT-8 cell monolayers with each purified subdomains proteins (40 µg/well) prior to infecting with *L. monocytogenes*. Data revealed that the number of adhered *L. monocytogenes* cells recovered from N2-pretreated HCT-8 cells was 1.77±0.05 log cfu/well and F-LAP-treated cells had 2.32±0.03 log cfu/well while all the other treatments including an untreated controls had bacterial counts of approximately 5.5 log cfu/ml (Fig 5). These data indicate that preoccupation of Hsp60 receptor on HCT-8 cells by N2 and also the F-LAP prevented *L. monocytogenes* from interacting with the monolayer. Collectively, these results clearly demonstrate that N2 is the primary LAP subdomain responsible for epithelial cell binding.

## Discussion

Many housekeeping enzymes act as “moonlighting proteins,” performing more than 1 function and playing a role in the bacterial virulence mechanism [34]. Examples include α-enolase of *Streptococcus pneumonia* [35], *S. pyogenes* [36], and *Mycoplasma fermentans* [37]; glyceraldehyde-3-phosphate dehydrogenase (GADPH) of *S. pyogenes* [36], *S. gordonii* [38], *E. coli* O157:H7 [39], and *L. monocytogenes* [40]; murein hydrolase (p60) of *L. monocytogenes* [41], alcohol acetaldehyde dehydrogenase (AAD or LAP) of *L. monocytogenes* [26], and *Entamoeba histolytica* [42]; and malate synthase of *Mycobacterium tuberculosis* [43]. These proteins are also known as “anchorless adhesins,” since no known cell surface anchoring mechanisms have been elucidated for these molecules [29]. As expected, these diverse classes of virulence factors interact with different host receptors. For instance, malate synthase binds to host laminin [43], enolase to plasminogen [37], [44], GADPH to plasmin [45], and AAD (LAP) to Hsp60 [26], [30].

In some instances, host receptors for these virulence factors and binding interactions to their respective receptors have been studied [29], [43], but the domains of enzymes participating in these interactions are poorly understood. We recently demonstrated that *L. monocytogenes* can cross the epithelial barrier through a paracellular route via an InlA-independent mechanism requiring the interaction of LAP and Hsp60 on the host cell surface [31]. Moreover, preexposure of intestinal cells to a low dosage of *L. monocytogenes* cells enhance host Hsp60 expression, promoting even greater LAP-mediated epithelial translocation and signifying the importance of the LAP–Hsp60 interaction in the infection process [31]. Thus, the goal of this study was to determine which LAP subdomain(s) interact with Hsp60. This information is important in the development of strategies to block LAP-domain function in controlling *L. monocytogenes* infections in high-risk populations.

LAP consists of 866 amino acids, has a pI of 6.58, and is rich in alanine (11%). Among the 4 subdomains tested for Hsp60 interaction, N2 is the smallest subdomain consisting of 188 amino acids, from Gly_224_–Gly_411_. The N2 domain is rich in lysine residues (11.7%) and along with arginine contains 25 positively charged residues with a calculated pI of 7.01, whereas N1, C1, and C2 contain 7.6%, 9.3%, and 6.4% lysine, respectively. Interestingly, p60, a muramidase that aids in adhesion to fibroblast cells, is also a lysine-rich protein [41], [46], similar to the LAP N2 subdomain. Although the N2 subdomain does not contain the characteristic Thr–Asn repeats of p60 [46], the presence of positively charged lysine residues in the N2 subdomain may play a role for interaction with receptor Hsp60 [47]. Likewise, fibronectin binding protein (Fbp) of *S. aureus* that interacts with Hsp60 [48] is also lysine-rich, containing 9.2% lysine residues, providing circumstantial evidence in support of our observation that the N2 subdomain, also rich in lysine residues, shows significant Hsp60 interaction and promotes adhesion to epithelial cells. Additionally, the role of lysine-rich proteins in promoting interaction with the host plasminogen has been well characterized in several bacteria and yeast [49], [50]. A lysine- and glutamic acid-rich protein 1 (KERP1) aids *E. histolytica* interaction with enterocyte-like Caco-2 cells [51]. Structurally, KERP1 is similar to LAP; it does not contain a signal peptide, transmembrane and/or glycosyl phosphoinositol (GPI) anchoring domains, and its trafficking to the plasma membrane is not well understood [52].

Through visual inspection of the LAP structure model–derived ModBase, 3 N-terminal ALDH region subdomains were identified. These subdomains include N1 (Met_1_–Pro_223_), N2 (Gly_22 4_–Gly_411_), and N3 (Gly_412_–Pro_464_). Two subdomains were identified in the C-terminal ADH region, including C1 (Pro_465_–Val_648_) and C2 (Pro_649_–Val_866_) (Fig. 1). Four LAP subdomains (N1, N2, C1, C2) were successfully cloned and expressed in a prokaryotic expression system. All processed and purified subdomain proteins were found to be stable (Fig. 2A), and were used in Hsp60 and epithelial cell binding assays. A less structured region, N3 possessing 52 amino acid residues (Fig 1), was thought to be structurally incompetent to interact independently with Hsp60, thus it was coexpressed with the C1 domain. The N2 subdomain was shown by the ligand overlay assay to interact with Hsp60. To further corroborate this result, an immunofluorescence assay was performed to test the ability of each subdomain to bind Hsp60. The N2 subdomain showed significantly higher Hsp60 binding than the other subdomains (*P*<0.05). Additionally, *K*_D_ values for N2 and F-LAP binding to Hsp60 were comparable and in agreement with our previous report [27]. Furthermore, bead-based epithelial cell binding assay and inhibition assay confirmed that the N2 subdomain is primarily responsible for binding to Hsp60 in the human ileocecal epithelial HCT-8 cell line, which previously showed strong LAP-mediated *L. monocytogenes* adhesion [24]. In all binding experiments, N2 subdomain appeared to display greater binding than the F-LAP (Fig 2 and 3) even though the same amount of protein was used for both. We speculate that this is because of higher number of N2 molecules per unit microgram than the full-length LAP, since N2 is significantly smaller (only 21 kDa) than LAP (104 kDa). To examine, if that was the case, we adjusted the protein concentrations to have equal number of molecules for each (supplementary data) and used in bead (fluospheres) binding experiment. Data show that both N2 (1363.8±101.6) and F-LAP (1377.1±99.5) - coated fluospheres had equivalent binding to HCT-8 cells (Fig S1). Collectively, these data strongly indicate that the N2 subdomain (Gly_224_–Gly_411_) in the LAP ALDH region contains the Hsp60 binding site.

In conclusion, we have provided evidence that the N2 subdomain (Gly_224_–Gly_411_) in the ALDH region of LAP is the site of initial Hsp60 receptor interaction. Interaction of the N2 subdomain with Hsp60 may lead to further downstream signaling events in intestinal cells [53], promoting adhesion and epithelial barrier crossing via the paracellular route by transepithelial translocation [31] in conjunction with other virulence factors [2]. The Hsp60 domain(s) that interacts with the LAP N2 subdomain is still unknown. Identification of the interaction domain will increase our understanding of the complex interaction between this housekeeping enzyme and chaperonin important for adhesion and transepithelial translocation of *L. monocytogenes* to intestinal epithelial cells.

## Materials and Methods

### Bacteria, Plasmid, and Media

Bacterial strains used included *L. monocytogenes* F4244 and *E. coli* DH5α (Life Technologies, Carlsbad, CA) for cloning of *lap* subdomains, and *E. coli* BL21(DE3) (Novagen, Gibbstown, NJ) for protein expression. *L. monocytogenes* was cultured in brain heart infusion broth (Becton Dickinson [BD], Sparks, MD) and *E. coli* cells were cultured in LB broth (BD). pGEMT-easy (Promega, Fitchburg, WI) and pET-32a (Novagen) vectors were used for cloning and expression of LAP domains. Ampicillin (50 µg/mL; Sigma, St. Louis, MO) was added to *E. coli* cultures containing the plasmids pGEMT-easy or pET-32a for selection.

### LAP Structure and Domain Prediction

LAP structural models were calculated using ModBase [32]. LAP contains 2 functional domains, an NAD-dependent N-terminal ALDH and a C-terminal ADH containing an iron binding site [27]. The predicted N-terminal domain structure is based on the nonphosphorylating GAPDH crystal structure (PDB ID: 1KY8) [54], and the C-terminal domain is based on the lactaldehyde reductase crystal structure (PDB ID:1RRM) [55]. The sequence identity of LAP and 1KY8 is 25.6 % while LAP and 1RRM is 35.5 %. ModBase provided a model score of 1.0 for both structure models, indicating the reliability of the predicted structures. Based on visual inspection of the predicted models, LAP is partitioned into 5 independent subdomains; N1 (Met_1_–Pro_223_), N2 (Gly_224_–Gly_411_), N3 (Gly_412_–Pro_464_), C1 (Pro_465_–Val_648_), and C2 (Pro_649_–Val_866_) (Fig. 1). A less-structured region, N3, was expressed with C1 domain and sequence boundaries were revised to be N1 (Met_1_–Pro_223_), N2 (Gly_224_–Gly_411_), C1 (Gly_412_–Val_648_), and C2 (Pro_649_–Val_866_). Due to the fact that the N-terminal ALDH and C-terminal ADH structural domains are modeled separately, we could not determine if N2 is in contact with C1 or C2.

### Cloning and Expression of LAP Subdomains

DNA encoding the LAP N1, N2, C1, and C2 were amplified using the primers listed in Table 1. *Bam*H1 and *Xho*1 restriction enzyme sites were added to the forward and reverse primers used to amplify the target *lap* subdomains. Amplified PCR products were cloned into the pGEMT-easy vector (Promega) and designated as pGEMT-N1, pGEMT-N2, pGEMT-C1, and pGEMT-C2. Clones were subsequently digested with *BamH*1 and *Xho*1 and inserted into a dephosphorylated pET-32a expression vector, resulting in pN1 LAP, pN2 LAP, pC1 LAP, and pC2 LAP. Presence of the insert was confirmed by restriction digestion, PCR, and sequencing at the Purdue University Genomics Facility.

**Table 1:** Primer sequences and LAP subdomain descriptions.

Primers	Primer sequences(5′ 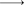 3′)	DNA base pairs	Predicted LAP protein
N1-F *Bam*H1	CGC GGA TCC a ATG GCA ATT AAA G	1–669 bp	23.8 kDa(Met_1_–Pro_223_)
N1-R *Xho*1	CCG CTC GAG b TTA TGG TCC AAC ACC		
N2- F *Bam*H1	CGC GGA TCC a GGT AAC GTA CCA GC	670–1233 bp	21.4 kDa(Gly_224_– Gly_411_)
N2- R *Xho*1	CCG CTC GAG b TTA GCC TTG TGC GCT TGG		
C1-F *Bam*H1	CGC GGA TCC a ATC GGT GAC AT	1237–1944 bp	27.7 kDa(Gly_412_–Val_648_)
C1- R *Xho*1	CCG CTC GAG b TTA AAC AGT AGT TAC		
C2- F*Bam*H1	CGC GGA TCC a CCA GCA CAC ATT AC	1945–2601 bp	25.6 kDa(Pro_649_–Val_866_)
C2- R *Xho*1	CCG CTC GAG b TCA AAC ACC TTT G		

a *BamH*1 site.

b *Xho*1 site.

### Purification of LAP Subdomains

*E. coli* BL 21(DE3) containing *lap* subdomain plasmids were grown in 250–500 mL of LB broth containing ampicillin (50 µg/mL). Protein overexpression (OD_600_ = 0.4) was induced with 0.2 mM IPTG (Sigma) followed by an additional 10 h of incubation at 25°C in a shaker incubator (New Brunswick Scientific). Cultures were then centrifuged (10,000 *g*, 10 min) and pellets were stored at −80°C overnight in 1× binding buffer from the His·Bind purification kit (EMD Chemicals, Gibbstown, NJ). Cell pellets were lysed by sonication (5 cycles, 30 s each cycle on ice, Branson Sonifier, Danbury, CT), centrifuged (16,000 *g*, 15 min), and the supernatants were collected. N-terminal His-tagged LAP subdomains were purified using the HisTrap, HP prepacked columns (G.E. Helathcare) and His·Bind resin kit following the manufacturer's instructions (EMD Chemicals). Protein-containing elutes were dialyzed using 12 kDa MWCO of dialysis tubing (Millipore, Billerica, MA), and protein concentration was estimated using the bicinchoninic acid (BCA) method (Pierce, Rockford, IL). Purity was assessed by SDS-PAGE (7.5% acrylamide gel), and purified proteins were used in all experiments. Preparation and purification of recombinant full-length LAP has been described previously [26].

### Ligand Overlay Assay for LAP Subdomain Binding to Hsp60

Purified LAP subdomains (∼8 µg each) were first separated by SDA-PAGE (7.5% Acrylamide gel) and proteins were then transferred to Immobilon P membranes (Millipore) [30]. Membranes were blocked using 5% non-fat dry milk and 0.05% Tween-20 at room temperature for 2 h, washed 3X with 20 mM phosphate buffered saline (pH 7.0) containing 0.5% Tween 20 (PBST) for 15 min each at room temperature. Membranes were then reacted with purified Hsp60 (4.8 µg/ml; Enzo Life Sciences) in PBS overnight at 4°C and subsequently washed three times with PBST for 15 min at room temperature before adding primary antibody. Membranes were reacted with mouse anti-Hsp60 MAb (1∶2000 dilution; Enzo Life Sciences) for 1.5 h at 37°C, washed (3X), and reacted with HRP-conjugated anti-mouse antibody (1:4000 dilutions; Jackson Immunologicals), developed using Pierce enhanced chemiluminescence substrate (Thermo Scientific) on X-ray film [31].

### Immunofluorescence Assay to Determine Binding Kinetics of LAP Subdomain Interaction with Hsp60

Each purified protein was resuspended in 0.05 M sodium carbonate coating buffer, pH 9.6, over a concentration range of 62.5–500 ng/100 µL, immobilized in 96-well Immulon 4HBX plates (Thermo Scientific, Waltham, MA), and stored at 4°C for 48 h. Before immobilization, the plate wells were first blocked with 3% BSA (Sigma) in PBST and then sequentially reacted with Hsp60 (100 µL of 2.4 µg/mL; Enzo Life Sciences), anti-Hsp60 antibody (100 µL of 0.87 µg/mL; Enzo Life Sciences), and anti-mouse HRP-conjugated antibody (100 µL of 0.5 µg/mL; Jackson Immunologicals). For all steps, plates were held at RT for 1.5 h and washed 3 times with PBST between steps. Finally 100 µL of Super Red, a fluorescent substrate (10-acetyl-3,7-dihydroxyphenoxazine; Virolabs, Chantilly, VA), was added to each well and fluorescence was measured (Ex: 540 nm; Em: 600 nm) using a Spectramax fluorescent reader (Gemini, Sunnyvale, CA) every 5 min for 60 min. To determine nonspecific protein binding, control reactions without LAP subdomains, Hsp60, and anti-Hsp60 were included. Fluorescent readings obtained from these controls were subtracted from the test results to obtain true binding levels. A 1-site binding equation included in the Prism 3 software (Graphpad) was used to determine *K*_D_ values of binding interactions.

### Adherence of Peptide-coated Microspheres to Epithelial Cells

Fifty microliters of sulphate-modified microsphere (fluospheres; 1.0 µm avg diameter, Invitrogen) was washed in 250 µL of HEPES binding buffer (HBB; 20 mM HEPES buffer in water, pH 7.2), centrifuged (14,000 *g*, 10 min), resuspended in HBB containing either 0.1% BSA (negative control) or 160 µg/mL of each purified recombinant proteins and incubated for 1 h at 37°C [56]. Beads were centrifuged once and the amounts of bound proteins were determined by BCA protein assay (Pierce). Amount of bound protein was calculated by subtracting the amount of unbound protein in the supernatant from the amount added. Microspheres were then resuspended in 0.1% BSA to block unbound surfaces, washed 4X with 10-fold volumes of HBB, and resuspended in HBB to achieve 10^9^ beads/mL.

Human ileocecal epithelial HCT-8 cells (CCL-244, ATCC) were grown in 96-well tissue culture plates until confluent monolayers were formed (∼4 days) and wells were washed once with 200 µl/well of PBS containing 0.5% BSA (PBSS) (Sigma). An 180-µl volume per well of DMEM-10F (Dulbecco's modified Eagle's medium containing 10% fetal bovine serum; Gibco, Carlsbad, CA) and 20 µl per well of diluted protein-bound fluosphere suspension (final ratio of cells to spheres was approximately 1∶100) were added to the monolayers. The diluted fluosphere suspensions were sonicated for 3 min to disrupt clumps prior to addition to the center of the wells. Blank wells did not receive any fluospheres. The sides of the plates were then tapped gently to mix and incubated for 2 h at 37°C under 5% CO_2_. Wells were then washed five times with 200 µl/well with PBSS, allowed to air dry before reading in the Spectramax spectrofluorometer at 485/538 nm [33]. Fluosphere binding to HCT-8 cells was also examined under fluorescence microscope (Leica, Wetzlar, Germany) equipped with Spot software (Sterling Heights, MI, USA) after counter staining the cell monolayers with Giemsa stain (Gibco).

To further demonstrate inhibition of F-LAP and N2 coated fluospheres binding to epithelial cells, we pretreated the HCT-8 cells with anti-Hsp60 monoclonal antibody to block surface Hsp60 as described before [30]. Briefly, HCT-8 confluent monolayers in 96-well tissue culture plates were washed once, treated with anti-Hsp60 MAb (1 µg/well; Enzo Life Sciences) for 1 h at 37°C, washed (5X), exposed to protein coated fluospehers (2 h) and the fluorescent reading was measured as above.

### Inhibition of *L. monocytogenes* Adhesion to Epithelial Cells by Recombinant Proteins

HCT-8 cells were grown in 24-well plates until the confluent monolayers were formed (∼4 days). The wells were then treated with 160 µg/ml of each purified recombinant proteins (F-LAP, N1, N2, C1, C2) or BSA (control) incubated for 1 h at 37°C. The wells were then washed three times with Cell-PBS (137 mM NaCl, 5.4 mM KCl, 3.5 mM Na_2_HPO_4_, 4.4 mM NaH_2_PO_4_, 11 mM glucose, pH 7.2). Freshly grown *L. monocytogenes* cultures were washed and resuspended in DMEM-10F and then added to cell monolayers at a multiplicity of infection (MOI) of 10. To measure bacterial adhesion, monolayers were washed after 1 h of infection, and adherent bacteria were enumerated by plating on BHI agar as described [31].

### Statistical Analysis

The SAS statistical software package (SAS Institute Inc., Cary, NC) was used to analyze immunofluorescence assay data. Significant difference was calculated at *P*<.05 and Tukey's studentized range test was used to group LAP domains based on their affinity to Hsp60. A pairwise Student's *t* test was used to analyze HCT-8 cell binding to beads coated with different LAP domains at *P*<0.05.

## Supporting Information

Figure S1 **Binding of fluospheres coated with equivalent number of molecules of LAP subdomain to HCT-8 cell monolayers.** Protein amounts for each subdomain were adjusted to contain equivalent number of molecules before immobilizing on beads. Data are average of three experiments analyzed in duplicate with SEM. Bars marked with letters (a,b) are significantly different at P<0.05.
(TIF)

## References

[1] Vazquez-Boland JA, Kuhn M, Berche P, Chakraborty T, Dominguez-Bernal G (2001). *Listeria* pathogenesis and molecular virulence determinants.. Clin Microbiol Rev.

[2] Pizarro-Cerda J, Cossart P (2006). Subversion of cellular functions by *Listeria monocytogenes*.. J Pathol.

[3] Cossart P, Pizarro-Cerdá J, Lecuit M (2003). Invasion of mammalian cells by *Listeria monocytogenes*: functional mimicry to subvert cellular functions.. Trends Cell Biol.

[4] Mengaud J, Ohayon H, Gounon P, Mege RM, Cossart P (1996). E-cadherin is the receptor for internalin, a surface protein required for entry of *L. monocytogenes* into epithelial cells.. Cell.

[5] Schubert WD, Urbanke C, Ziehm T, Beier V, Machner MP (2002). Structure of internalin, a major invasion protein of *Listeria monocytogenes*, in complex with its human receptor E-cadherin.. Cell.

[6] Pizarro-Cerda J, Cossart P (2006). Bacterial adhesion and entry into host cells.. Cell.

[7] Ireton K (2007). Entry of the bacterial pathogen *Listeria monocytogenes* into mammalian cells.. Cell Microbiol.

[8] Pentecost M, Kumaran J, Ghosh P, Amieva MR (2010). *Listeria monocytogenes* Internalin B activates junctional endocytosis to accelerate intestinal invasion.. PLoS Pathog.

[9] Braun L, Ghebrehiwet B, Cossart P (2000). gC1q-R/p32, a C1q-binding protein, is a receptor for the InlB invasion protein of *Listeria monocytogenes*.. EMBO J.

[10] Shen Y, Naujokas K, Park M, Ireton K (2000). InlB-dependent internalization of *Listeria* is mediated by the Met receptor tyrosine kinase.. Cell.

[11] Disson O, Grayo S, Huillet E, Nikitas G, Langa-Vives F (2008). Conjugated action of two species-specific invasion proteins for fetoplacental listeriosis.. Nature.

[12] Sabet C, Toledo-Arana A, Personnic N, Lecuit M, Dubrac S (2008). The *Listeria monocytogenes* virulence factor InlJ is specifically expressed in vivo and behaves as an adhesin.. Infect Immun.

[13] Cabanes D, Sousa S, Cebria A, Lecuit M, Garcia-del Portillo F (2005). Gp96 is a receptor for a novel *Listeria monocytogenes* virulence factor, Vip, a surface protein.. EMBO J.

[14] Milohanic E, Jonquieres R, Glaser P, Dehoux P, Jacquet C (2004). Sequence and binding activity of the autolysin-adhesin Ami from epidemic *Listeria monocytogenes* 4b.. Infect Immun.

[15] Dramsi S, Bourdichon F, Cabanes D, Lecuit M, Fsihi H (2004). FbpA, a novel multifunctional *Listeria monocytogenes* virulence factor.. Mol Microbiol.

[16] Gilot P, Andre P, Content J (1999). *Listeria monocytogenes* possesses adhesins for fibronectin.. Infect Immun.

[17] Abachin E, Poyart C, Pellegrini E, Milohanic E, Fiedler F (2002). Formation of D-alanyl-lipoteichoic acid is required for adhesion and virulence of *Listeria monocytogenes*.. Mol Microbiol.

[18] Xayarath B, Marquis H, Port GC, Freitag NE (2009). *Listeria monocytogenes* CtaP is a multifunctional cysteine transport-associated protein required for bacterial pathogenesis.. Mol Microbiol.

[19] Reis O, Sousa S, Camejo A, Villiers V, Gouin E (2010). LapB, a novel *Listeria monocytogenes* LPXTG surface adhesin, required for entry into eukaryotic cells and virulence.. J Infect Dis.

[20] Bierne H, Cossart P (2007). *Listeria monocytogenes* surface proteins: from genome predictions to function.. Microbiol Mol Biol Rev.

[21] Lecuit M, Vandormael-Pournin S, Lefort J, Huerre M, Gounon P (2001). A transgenic model for listeriosis: Role of internalin in crossing the intestinal barrier.. Science.

[22] Lecuit M, Ohayon H, Braun L, Mengaud J, Cossart P (1997). Internalin of *Listeria monocytogenes* with an intact leucine-rich repeat region is sufficient to promote internalization.. Infect Immun.

[23] Lebrun M, Mengaud J, Ohayon H, Nato F, Cossart P (1996). Internalin must be on the bacterial surface to mediate entry of *Listeria monocytogenes* into epithelial cells.. Mol Microbiol.

[24] Jaradat ZW, Wampler JW, Bhunia AW (2003). A Listeria adhesion protein-deficient *Listeria monocytogenes* strain shows reduced adhesion primarily to intestinal cell lines.. Med Microbiol Immunol.

[25] Pandiripally VK, Westbrook DG, Sunki GR, Bhunia AK (1999). Surface protein p104 is involved in adhesion of *Listeria monocytogenes* to human intestinal cell line, Caco-2.. J Med Microbiol.

[26] Kim KP, Jagadeesan B, Burkholder KM, Jaradat ZW, Wampler JL (2006). Adhesion characteristics of Listeria adhesion protein (LAP)-expressing *Escherichia coli* to Caco-2 cells and of recombinant LAP to eukaryotic receptor Hsp60 as examined in a surface plasmon resonance sensor.. FEMS Microbiol Lett.

[27] Jagadeesan B, Koo O-K, Kim K-P, Burkholder KM, Mishra KK (2010). LAP, an alcohol acetaldehyde dehydrogenase enzyme in *Listeria* promotes bacterial adhesion to enterocyte-like Caco-2 cells only in pathogenic species.. Microbiology.

[28] Burkholder KM, Kim K-P, Mishra K, Medina S, Hahm B-K (2009). Expression of LAP, a SecA2-dependent secretory protein, is induced under anaerobic environment.. Microbes Infect.

[29] Pancholi V, Chhatwal GS (2003). Housekeeping enzymes as virulence factors for pathogens.. Int J Med Microbiol.

[30] Wampler JL, Kim KP, Jaradat Z, Bhunia AK (2004). Heat shock protein 60 acts as a receptor for the *Listeria* adhesion protein in Caco-2 cells.. Infect Immun.

[31] Burkholder KM, Bhunia AK (2010). *Listeria monocytogenes* uses Listeria Adhesion Protein (LAP) to promote bacterial transepithelial translocation and induces expression of LAP receptor Hsp60.. Infect Immun.

[32] Pieper U, Eswar N, Braberg H, Madhusudhan MS, Davis FP (2004). MODBASE, a database of annotated comparative protein structure models, and associated resources.. Nucl Acids Res.

[33] Romero-Steiner S, Caba J, Rajam G, Langley T, Floyd A (2006). Adherence of recombinant pneumococcal surface adhesin A (rPsaA)-coated particles to human nasopharyngeal epithelial cells for the evaluation of anti-PsaA functional antibodies.. Vaccine.

[34] Henderson B, Allan E, Coates AR (2006). Stress wars: the direct role of host and bacterial molecular chaperones in bacterial infection.. Infect Immun.

[35] Bergmann S, Rohde M, Chhatwal GS, Hammerschmidt S (2001). alpha-Enolase of *Streptococcus pneumoniae* is a plasmin(ogen)-binding protein displayed on the bacterial cell surface.. Mol Microbiol.

[36] Pancholi V, Fischetti VA (1992). A major surface protein on group A streptococci is a glyceraldehyde-3-phosphate-dehydrogenase with multiple binding activity.. J Exp Med.

[37] Yavlovich A, Rechnitzer H, Rottem S (2007). Alpha-enolase resides on the cell surface of *Mycoplasma fermentans* and binds plasminogen.. Infect Immun.

[38] Nelson D, Goldstein JM, Boatright K, Harty DWS, Cook SL (2001). pH-regulated secretion of a glyceraldehyde-3-phosphate dehydrogenase from *Streptococcus gordonii* FSS2: Purification, characterization, and cloning of the gene encoding this enzyme.. J Dental Res.

[39] Egea L, Aguilera L, Gimenez R, Sorolla MA, Aguilar J (2007). Role of secreted glyceraldehyde-3-phosphate dehydrogenase in the infection mechanism of enterohemorrhagic and enteropathogenic *Escherichia coli*: Interaction of the extracellular enzyme with human plasminogen and fibrinogen.. Int J Biochem Cell Biol.

[40] Alvarez-Dominguez C, Madrazo-Toca F, Fernandez-Prieto L, Vandekerckhove J, Pareja E (2008). Characterization of a *Listeria monocytogenes* protein interfering with Rab5a.. Traffic.

[41] Wuenscher MD, Kohler S, Bubert A, Gerike U, Goebel W (1993). The *iap* gene of *Listeria monocytogenes* is essential for cell viability, and its gene product, p60, has bacteriolytic activity.. J Bacteriol.

[42] Yang W, Li E, Kairong T, Stanley SL (1994). *Entamoeba histolytica* has an alcohol dehydrogenase homologous to the multifunctional *adhE* gene product of *Escherichia coli*.. Mol Biochem Parasitol.

[43] Kinhikar AG, Vargas D, Li H, Mahaffey SB, Hinds L (2006). *Mycobacterium tuberculosis* malate synthase is a laminin-binding adhesin.. Mol Microbiol.

[44] Pancholi V, Fontan P, Jin H (2003). Plasminogen-mediated group A streptococcal adherence to and pericellular invasion of human pharyngeal cells.. Microb Pathog.

[45] Bergmann S, Rohde M, Hammerschmidt S (2004). Glyceraldehyde-3-phosphate dehydrogenase of *Streptococcus pneumoniae* is a surface-displayed plasminogen-binding protein.. Infect Immun.

[46] Kohler S, Leimeister-Wachter M, Chakraborty T, Lottspeich F, Goebel W (1990). The gene coding for protein p60 of Listeria monocytogenes and its use as a specific probe for Listeria monocytogenes.. Infect Immun.

[47] Koba H, Okuda K, Watanabe H, Tagami J, Senpuku H (2009). Role of lysine in interaction between surface protein peptides of *Streptococcus gordonii* and agglutinin peptide.. Oral Microbiology and Immunology.

[48] Dziewanowska K, Carson AR, Patti JM, Deobald CF, Bayles KW (2000). Staphylococcal fibronectin binding protein interacts with heat shock protein 60 and integrins: Role in internalization by epithelial cells.. Infect Immun 68:.

[49] Lahteenmaki K, Edelman S, Korhonen TK (2005). Bacterial metastasis: the host plasminogen system in bacterial invasion.. Trends Microbiol.

[50] Stie J, Bruni G, Fox D (2009). Surface-associated plasminogen binding of *Cryptococcus neoformans* promotes extracellular matrix invasion.. Plos One.

[51] Seigneur M, Mounier J, Prevost MC, Guillen N (2005). A lysine- and glutamic acid-rich protein, KERP1, from *Entamoeba histolytica* binds to human enterocytes.. Cell Microbiol.

[52] Santi-Rocca J, Weber C, Guigon G, Sismeiro O, Coppee JY (2008). The lysine- and glutamic acid-rich protein KERP1 plays a role in *Entamoeba histolytica* liver abscess pathogenesis.. Cell Microbiol.

[53] Ranford JC, Coates ARM, Henderson B (2000). Chaperonins are cell-signalling proteins: the unfolding biology of molecular chaperones.. Expert Rev Mol Med.

[54] Pohl E, Brunner N, Wilmanns M, Hensel R (2002). The crystal structure of the allosteric non-phosphorylating glyceraldehyde-3-phosphate dehydrogenase from the hyperthermophilic archaeum *Thermoproteus tenax*.. J Biol Chem.

[55] Kumaran D, Swaminathan S (2011). Crystal structure of lactaldehyde reductase.. http://www.rcsb.org/pdb/explore.do?structureId=1RRM.

[56] Ainscough SL, Feigl B, Malda J, Harkin DG (2009). Discovery and characterization of IGFBP-mediated endocytosis in the human retinal pigment epithelial cell line ARPE-19.. Exp Eye Res.

