# Supplementary figures and images for: N-Terminal Gly224–Gly411 Domain in Listeria Adhesion Protein Interacts with Host Receptor Hsp60
Source: PLoS One. 2011 Jun 29;6(6):e20694. doi: 10.1371/journal.pone.0020694 (PMC3126804; doi:10.1371/journal.pone.0020694)

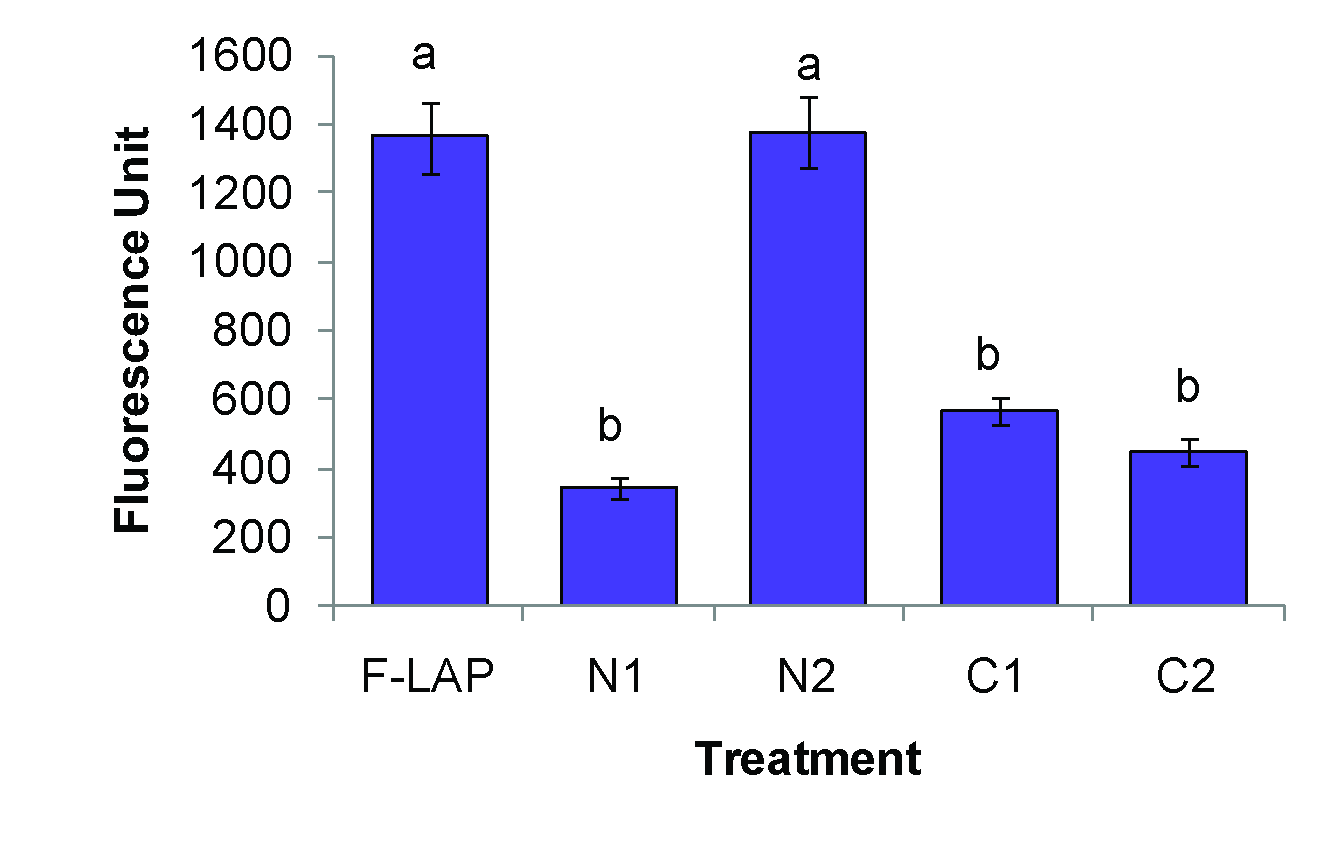

Supplement: Figure S1 — Binding of fluospheres coated with equivalent number of molecules of LAP subdomain to HCT-8 cell monolayers. Protein amounts for each subdomain were adjusted to contain equivalent number of molecules before immobilizing on beads. Data are average of three experiments analyzed in duplicate with SEM. Bars marked with letters (a,b) are significantly different at P<0.05. (TIF) [file pone.0020694.s001.tif]
